# Establishing a Female Animal Model of Prediabetes Using a High-Carbohydrate, High-Fat Diet

**DOI:** 10.3390/cimb46110736

**Published:** 2024-11-03

**Authors:** Ayanda Nkosi, Reveshni Pather, Bongeka Mshengu, Andile Khathi, Phikelelani Ngubane

**Affiliations:** 1 School of Laboratory Medicine and Medical Sciences, University of KwaZulu-Natal, Westville, Durban 4000, South Africa; 216000895@stu.ukzn.ac.za (A.N.); 223029565@stu.ukzn.ac.za (B.M.); khathia@ukzn.ac.za (A.K.); 2 Department of Human Physiology, University of KwaZulu-Natal, Westville, Durban 4000, South Africa; 215011122@stu.ukzn.ac.za

**Keywords:** prediabetes, high-carbohydrate, high-fat diet, insulin resistance, impaired glucose tolerance

## Abstract

Prediabetes is a condition that often precedes the onset of type 2 diabetes and is characterized by moderate levels of insulin resistance. This condition is well established in male animal models for diabetes; however, few female models exist. There is accumulating evidence that sex variations affect the pathogenesis, treatment, and consequences of numerous diseases, such as type 2 diabetes. Therefore, we sought to develop a diet-induced prediabetic female animal model to better understand prediabetes development and its effects in females. Female Sprague Dawley rats were randomly allocated to one of two groups: the standard diet (SD) group fed a standard diet with normal drinking water, and the high-carbohydrate, high-fat (HCHF) group fed a high-carbohydrate and high-fat diet with drinking water supplemented with fructose. During induction, we measured food intake, body weight, body mass index (BMI), and oral glucose tolerance response (OGT). After the induction period, biochemical analyses were conducted to assess the levels of plasma leptin, ghrelin, insulin, and glycated hemoglobin (HbA1c). Glycogen concentrations were quantified in the liver and skeletal muscles. The HCHF diet-fed group presented higher body weight gain, food intake, and BMI levels, which were accompanied by elevated plasma insulin, ghrelin, and liver and skeletal muscle glycogen levels compared to the SD-fed group. In the HCHF diet-fed group, the HOMA-IR was above 1.9, suggesting the presence of moderate levels of insulin resistance. The OGT response was significantly higher in the HCHF-fed group versus the SD-fed group, suggesting impaired glucose tolerance, thus displaying the signs and symptoms of prediabetes. The HCHF diet with fructose led to the induction of prediabetes in female Sprague Dawley rats. This model could be used to investigate and outline the pathophysiological complications associated with prediabetes in females as a result of the prolonged ingestion of a high carbohydrate, high-fat diet with fructose. The development of this model could also serve as an effort to further bridge the gap regarding the inclusion of females in biomedical research, thus providing advancements in deriving better, specified treatment strategies for women.

## 1. Introduction

The rising prevalence of type 2 diabetes (T2DM) is considered an important global health concern [1]. Five hundred thirty-seven million individuals have been estimated to be living with diabetes, and T2DM represents 98% of global diabetes diagnoses, with greater incidence in men than in women [2,3]. However, at the time of type 2 diabetes diagnosis, women appear to face a larger risk factor burden [4]. The rapid development of the economy and urbanization has led to the growing prevalence of T2DM in many parts of the world, affecting the quality of life of many people and resulting in serious morbidity and premature mortality [5]. This is related to the increases in body mass index and fasting plasma glucose caused by the high consumption of unhealthy diets and the incidence of sedentary lifestyles [6].

Prediabetes is typically present before overt type 2 diabetes develops, which indicates impaired glucose metabolism, including impaired glucose tolerance (IGT) and impaired fasting glucose (IFG); these both signal blood glucose levels that are higher than normal but lower than the diabetes threshold [7]. According to estimates, 319 million individuals worldwide have prediabetes, with a higher prevalence in males than in females [8,9]. The oral glucose tolerance test (OGTT) is a common way to diagnose or screen for impaired glucose tolerance and T2DM [10]. It is also one method of assessing one’s apparent insulin sensitivity and insulin resistance [10]. The American Diabetes Association (ADA) has suggested the use of glycated hemoglobin (Hb1Ac) as a diagnostic test for diabetes, and it can also be utilized to identify prediabetes [11]. This test indicates a person’s typical blood glucose levels over the previous 2–3 months, and the greater the levels are, the higher the chance of acquiring issues associated with diabetes [12]. The Homeostatic Model Assessment of Insulin Resistance (HOMA-IR) has been routinely used to assess insulin sensitivity [13]. HOMA-IR readings below 1 show the highest insulin sensitivity levels, with levels above 1.9 suggesting the onset of insulin resistance and above 2.9 indicating significant insulin resistance [14].

Two hormones are mainly associated with controlling appetite and satiety to maintain energy homeostasis: ghrelin and leptin [15]. By inhibiting food intake and promoting weight reduction, leptin functions as a mediator in the long-term regulation of energy balance [15]. On the other hand, the fast-acting hormone ghrelin appears to be involved in meal initiation [16]. There is accumulating evidence that sex variations affect the pathogenesis, treatment, and consequences of numerous diseases, such as type 2 diabetes [17].

In our laboratory, we have previously established a diet-induced prediabetic animal model from the voluntary ingestion of a high carbohydrate, high-fat diet supplemented with fructose to better understand the progression of prediabetes [18]. Mostly, all studies reported on dietary manipulation and metabolism have been typically undertaken in male rodents [19]. Some contributing factors to this are concerns about the oestrus cycle’s potential effects on females’ metabolic parameters independent of dietary manipulation and the suggested protective role of female hormones on metabolic parameters [19]. There is, therefore, a paucity of literature on establishing prediabetic female animal models, which warrants the need to develop a diet-induced prediabetic female animal model to provide an improved understanding of the development and effects of prediabetes in females. Therefore, in this study, we used a high-carbohydrate, high-fat diet to establish a female rat model for prediabetes; the impacts of the prolonged ingestion of this diet on various biomarkers for prediabetes were assessed.

## 2. Materials and Methods

### 2.1. Animals and Housing

The study utilized 12 female Sprague Dawley rats that weighed 150–180 g. They were bred and kept in Makrolon polycarbonate metabolic cages (Techniplast, Labotec, South Africa) in the Biomedical Research Unit of the University of KwaZulu-Natal. The University of KwaZulu-Natal’s Animal Research Ethics Committee, which adheres to the values and standards of the Canadian Council on Animal Care, authorized all animal procedures and housing arrangements. The animals were kept under standard laboratory conditions for temperature and humidity in a 12-h day and 12-h night cycle. The noise levels were restricted to about 65 dB. The animals had unrestricted access to food and water. The University of KwaZulu-Natal’s Animal Research Ethics Committee reviewed and authorized all animal experiments (Ethical clearance number: (AREC/031/019D)). Close monitoring was performed for pain and discomfort using the UKZN institutional animal ethics committee’s humane endpoint documentation criteria. The animals were acclimatized to their new environment for 1 week before the commencement of the study. The animals were administered a normal diet (EPOL, Pretoria West, South Africa) and normal drinking water for acclimatization. A single-blind technique was followed to randomly divide the animals into experimental groups to prevent bias. The animals were divided into separate groups by the animal technician, who was not involved in the study. The animals were kept in specific groups throughout the experimental period and never mixed.

### 2.2. Induction of Prediabetes

After acclimatization, female Sprague Dawley rats were randomly assigned to the following diet-specific groups: the standard diet (SD) group, which received a standard rat chow diet and water (*n* = 6), and the high-carbohydrate, high-fat (HCHF) group, which received a high-carbohydrate, high-fat diet (AVI-Products (Pty) Ltd., Cato Ridge, South Africa) supplemented with water with 15% fructose (*n* = 6).

Using a previously reported protocol, the animals were induced with experimental prediabetes [18]. Briefly, prediabetes was induced in female Sprague Dawley rats by allowing the animals to consume a high-carbohydrate, high-fat (HCHF) diet with drinking water supplemented with 15% fructose for 36 weeks. Prediabetes was confirmed in the animals, using the American Diabetes Association criteria for prediabetes, which is defined by the presence of impaired glucose tolerance (IGT) between 7.8 and 11.0 mmol/L and/or impaired fasting glucose (IFG) between 6.1 and 6.9 mmol/L [20].

The sample size calculation was conducted to obtain the desired sample size necessary to induce prediabetes and its complications in a female Sprague Dawley rat model.

### 2.3. Experimental Design

**Figure 1:**
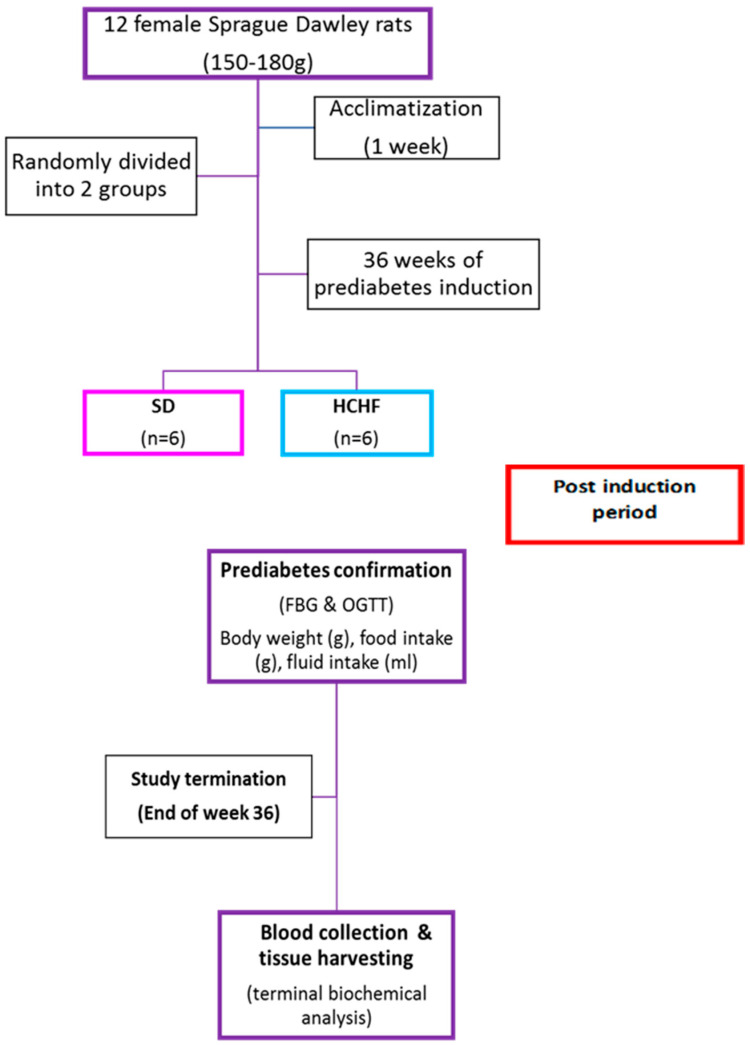
A diagrammatical depiction of the experimental design.

The following groups of female Sprague Dawley rats were randomly assigned (*n* = 6): the standard diet (SD) group received a standard rat chow diet with water (SD group), and the high-carbohydrate, high-fat (HCHF) group received a high-carbohydrate, high-fat diet with water supplemented with 15% fructose (HCHF group), (AVI-Products (Pty) Ltd., Cato Ridge, South Africa) (Figure 1). The composition of the high-carbohydrate, high-fat diet was customized, as seen in Table 1, and the percentage composition of the standard diet and high-carbohydrate, high-fat diet are seen in Table 2 (EPOL, Pretoria West, South Africa). The animal body weight, food intake, BMI, fasting blood glucose, and oral glucose tolerance (OGT) responses were all recorded every four weeks during the experimental period. 

### 2.4. Determination of Body Mass Index

The determination of BMI was measured from the ratio of the weight to the square of the length of each animal, as described in the established protocol [21]. The BMI was calculated as follows:

Body mass index **=** weight (g)/[length (cm)]^2^ (1)

### 2.5. Calculation of Food and Water Intake

The food and water intake levels were recorded every four weeks during the experimental period. The animals were placed in specialized metabolic cages to measure food and water intake for 24 h. Each animal was provided with 100 g of their respective diet and 100 mL of their respective water type. After 24 h, the remaining grams of food and the volume of water that the animals had not consumed were measured.

### 2.6. Oral Glucose Tolerance Response

Following carbohydrate loading (glucose concentration was 0.86 g/kg), an oral glucose tolerance test was carried out according to a set laboratory protocol to determine the animals’ glucose tolerance responses [22]. Briefly, the animals were fasted for 18 h, and at the end of the fasting period, the fasting blood glucose levels were measured (time 0). Carbohydrate loading was measured individually and administered according to the current weight of each animal at a concentration of 0.86 g/kg glucose. An 18-gauge gavage needle that is 38 mm in length, curved, and had a 21/4 mm ball end was used to load the animals with monosaccharide syrup (glucose; 0.86 g/kg) via oral gavage (Able Scientific, Canning Vale, Australia). Blood was drawn using the tail prick technique [23], and a OneTouch select glucometer (Lifescan, Mosta, Malta) was used to measure the glucose concentration in the blood. The glucose levels were assessed 15, 30, 60, and 120 min after carbohydrate loading.

### 2.7. Blood Collection and Tissue Harvesting

All the animals were sacrificed by decapitation at the end of week 36 using a sharpened guillotine. Blood was collected in pre-cooled EDTA tubes. After 15 min of centrifuging the blood at 4 °C, 503 g (Eppendorf centrifuge 5403, Hamburg, Germany), the plasma was collected and kept at −80 °C in a Bio Ultra freezer until it was required for biochemical analysis. Plasma samples were used to measure insulin, ghrelin, and leptin concentrations. Furthermore, the skeletal muscle and liver tissues were excised, weighed, and stored in pre-cooled Eppendorf containers and snap-frozen in liquid nitrogen before storage in a Bio Ultra freezer (Snijers Scientific, Tilburg, Holland) at −80 °C.

### 2.8. Biochemical Analysis

Using the appropriate rat ELISA kits, HbA1c, plasma leptin, ghrelin, and insulin concentrations were determined (Elabscience Biotechnology Co., Ltd., Wuhan, China) while following the manufacturer’s instructions. The micro-ELISA plates were coated with antibodies as part of the standard experimental protocol in the ELISA kits. The plasma samples were pipetted into the appropriate wells, followed by the immediate addition of the appropriate biotinylated detection antibody (50 μL). The samples were then incubated for 45 min at 37 °C, after which the unbound components were washed away with the supplied wash buffer. After washing, the wells were filled with 100 μL of Avidin-horseradish peroxidase (HRP), which was incubated at 37 °C for 30 min. After removing the unattached components with a second wash, the substrate reagent (90 μL) was applied to the wells. This was followed by a 15 min incubation period at 37 °C. Finally, a stop solution (50 μL) was applied to the micro-wells to stop the reaction and allow for appropriate measurements. The optical density at 450 nm was determined using a nano-spectrophotometer (BMG Labtech, Ortenburg, Germany). Glycated hemoglobin, plasma leptin, ghrelin, and insulin concentrations in the samples were extrapolated from their respective standard curves.

#### Glycogen Assay

Glycogen analysis was conducted in the liver and skeletal muscles following a previously established protocol [24]. Briefly, the harvested tissues were weighed and heated with potassium hydroxide (KOH) (30%, 2 mL) for 30 min at 100 °C. Immediately, 0.194 mL of 10% sodium tetraoxosulphate VI (Na_2_SO_4_) was added to the mixture to stop the reaction. When the mixture was allowed to cool, the glycogen precipitate was formed. A total of 200 μL of the cooled mixture with the precipitate was aspirated and mixed with ethanol (95%, 200 μL). The precipitated glycogen was pelleted, washed, and resolubilized in H_2_O (1 mL). Thereafter, 4 mL of anthrone (0.5 g dissolved in 250 mL of 95% sulphuric acid) was added and boiled for 10 min. After cooling, the absorbance was determined by using the Spectrostar Nano spectrophotometer (BMG Labtech, Ortenburg, LGBW Germany) at 620 nm.

### 2.9. Data Analysis

Statistical comparisons were performed with R statistical software (version 4.3.3). We utilized box plots to visually represent the distribution of the data. We assessed the normality of the data using the Shapiro test. To evaluate whether there were statistically significant differences between the groups, we utilized either a parametric or non-parametric test, depending on the distribution of the data. Since the data were collected from two independent samples, the HCHF and SD groups, we employed an independent two-sample *t*-test or its non-parametric counterpart, the Wilcoxon rank sum test, to assess statistical differences. We employed a non-parametric Friedman test to assess potential variations within the same groups across various time points (weeks 12, 24, and 36). Non-parametric data were reported as the median (interquartile range), and parametric data were reported as the mean and standard deviation (SD), with a 95% confidence interval (CI) for the mean. The statistical significance was defined as a *p*-value < 0.05.

## 3. Results

### 3.1. Fasting Blood Glucose

**Figure 2:**
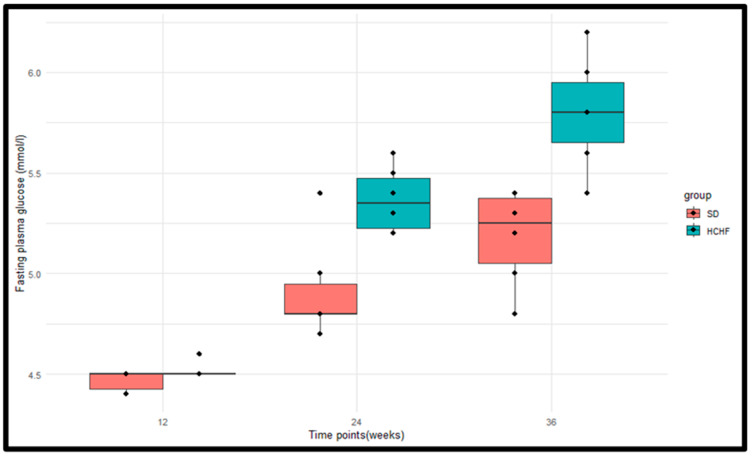
Boxplots illustrating the distributions of fasting plasma glucose levels in the SD group and the HCHF group at weeks 12, 24, and 36. The horizontal line within each box represents the median, while the box encompasses the interquartile range (IQR). The whiskers extend to the minimum and maximum values. The individual measurements are represented by the black dots.

The FBG values were measured for the standard diet SD group (*n* = 6) and the HCHF group (*n* = 6) at weeks 12, 24, and 36 of induction (Figure 2). The *p*-values obtained from the Wilcoxon rank sum test were <0.05 at weeks 24 and 36, indicating a significant difference in fasting plasma glucose levels between the SD and HCHF groups. There was no significant difference between the two groups at week 12 (Table 3). The *p*-values obtained from the Friedman test indicated that there were significant differences in the fasting plasma glucose within the HCHF group and the SD group (Table 4).

### 3.2. Oral Glucose Tolerance (OGT)

**Figure 3:**
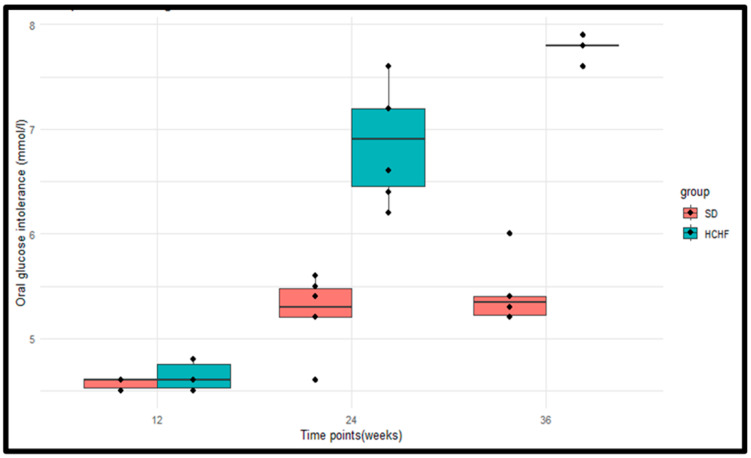
Boxplots illustrating the distributions of the post-prandial glucose concentrations of the SD group and the HCHF group at weeks 12, 24, and 36. The horizontal line within each box represents the median, while the box encompasses the interquartile range (IQR). The whiskers extend to the minimum and maximum values. The black dots represent the individual measurements.

The OGTT was measured in the SD group (*n* = 6) and the HCHF group (*n* = 6) at weeks 12, 24 and 36 of induction (Figure 3). The *p*-values obtained from the Wilcoxon rank sum test were <0.05 at weeks 24 and 26, indicating a significant difference between the SD and HCHF groups in the post-prandial glucose concentrations after 2 h. There were no significant differences between the two groups at week 12 (Table 5). The *p*-values obtained from the Friedman test indicated significant differences in the post-prandial glucose concentration within the HCHF group and the SD group (Table 6).

### 3.3. Food Intake

**Figure 4:**
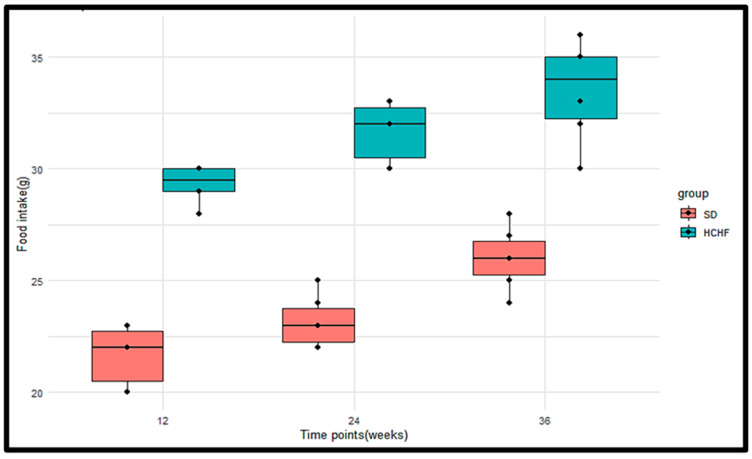
Boxplots illustrating the distributions of the food intake levels of the SD group and the HCHF group at weeks 12, 24, and 36. The horizontal line within each box represents the median, while the box encompasses the interquartile range (IQR). The whiskers extend to the minimum and maximum values. The black dots represent the individual measurements.

The food intake levels were measured in the SD group (*n* = 6) and the HCHF group (*n* = 6) at weeks 12, 24, and 36 of induction (Figure 4). The *p*-values obtained from the Wilcoxon rank sum test were <0.05 at weeks 12, 24, and 36, indicating a significant difference in the food intake levels between the SD group and the HCHF group, with the HCHF group having consistently higher food intake levels at weeks 12, 24 and 36 compared to the SD group (Table 7). The *p*-values obtained from the Friedman test indicated that there were significant differences in the food intake levels within the HCHF group and within the SD group (Table 8).

### 3.4. Body Weights

**Figure 5:**
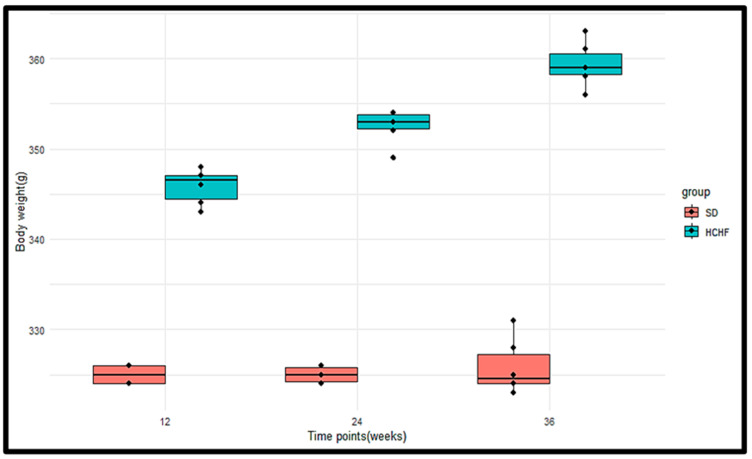
Boxplots illustrating the distributions of the SD and HCHF groups’ body weights at weeks 12, 24, and 36. The horizontal line within each box represents the median, while the box encompasses the interquartile range (IQR). The whiskers extend to the minimum and maximum values. The black dots represent the individual measurements.

The animal body weights were measured in the (SD) group (*n* = 6) and the HCHF group (*n* = 6) at weeks 12, 24, and 36 of induction (Figure 5). The *p*-values obtained from the Wilcoxon rank sum test were <0.05 at weeks 12, 24, and 36, indicating a significant difference in the body weights between the SD group and the HCHF group, with the HCHF group having consistently higher body weights at weeks 12, 24 and 36 compared to the SD group (Table 9). The *p*-values obtained from the Friedman test indicated that there were significant differences in the body weights within the HCHF group; however, there were no significant differences within the SD group (Table 10).

### 3.5. Body Mass Index

**Figure 6:**
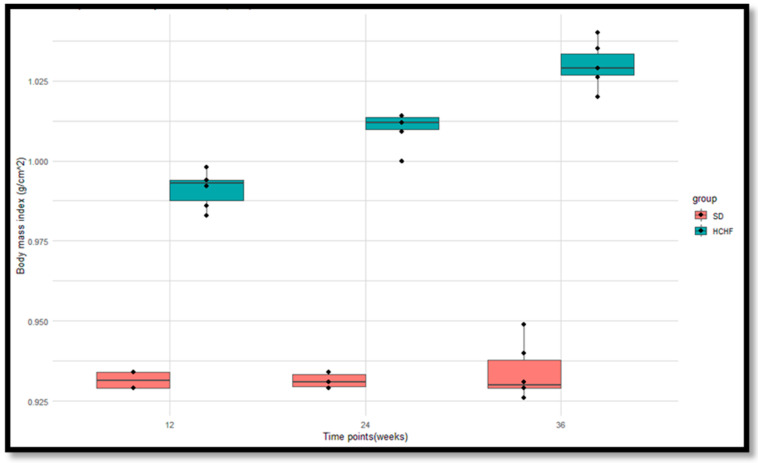
Boxplots illustrating the distributions of the BMIs of the SD group and the HCHF group at weeks 12, 24, and 36. The horizontal line within each box represents the median, while the box encompasses the interquartile range (IQR). The whiskers extend to the minimum and maximum values. The black dots represent the individual measurements.

The BMIs of the (SD) group (*n* = 6) and the HCHF group (*n* = 6) were calculated at weeks 12, 24, and 36 of induction (Figure 6). The *p*-values obtained from the Wilcoxon rank sum test were <0.05 at weeks 12, 24, and 26, indicating significant differences in the BMIs between the SD group and the HCHF group, with the HCHF group having consistently higher BMIs at weeks 12, 24 and 36 compared to the SD group (Table 11). The *p*-values obtained from the Friedman test indicate significant differences in the BMIs within the HCHF group; however, there are no significant differences within the SD group (Table 12).

### 3.6. Glycated Hemoglobin (HbA1c)

**Figure 7:**
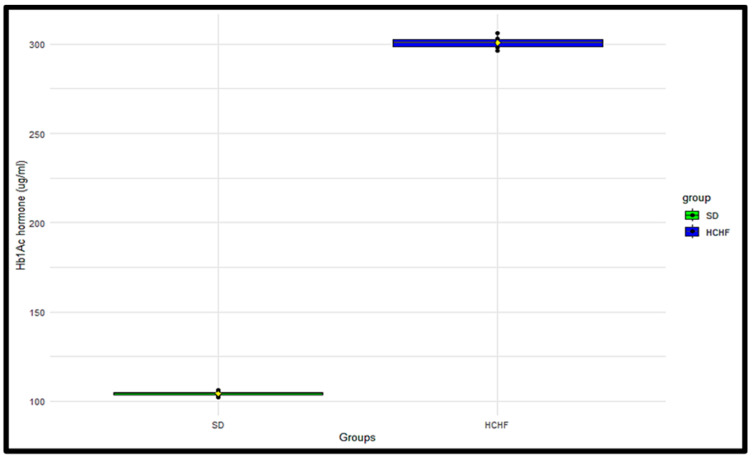
The terminal distributions of glycated hemoglobin concentrations in the SD and HCHF groups. The horizontal line within each box represents the median, while the box encompasses the interquartile range (IQR). The whiskers extend to the minimum and maximum values. The yellow diamond shape represents the mean. The black dots represent the individual measurements.

The HbA1c concentrations were measured terminally in the SD group (*n* = 6) and the HCHF group (*n* = 6) (Figure 7). The *p*-values obtained from the independent two-sample *t*-test were <0.05, indicating significant differences in the HbA1c concentrations between the SD group and the HCHF group, with the HCHF group having higher HbA1c concentrations compared to the SD group (Table 13).

### 3.7. Glycogen

**Figure 8:**
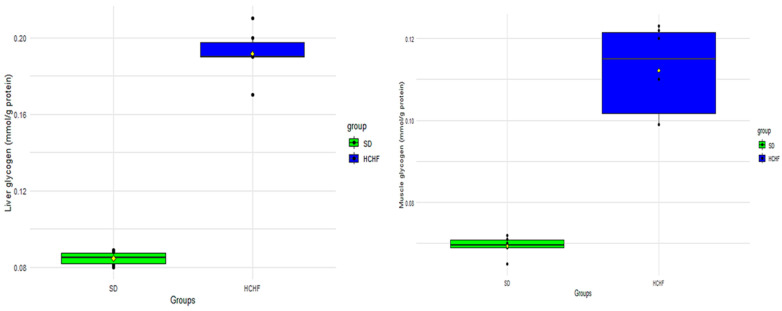
The terminal distributions of liver and skeletal muscle glycogen concentrations in the SD and HCHF groups. The horizontal line within each box represents the median, while the box encompasses the interquartile range (IQR). The whiskers extend to the minimum and maximum values. The yellow diamond shape represents the mean. The black dots represent the individual measurements.

The liver and skeletal muscle glycogen levels were measured terminally in the SD and HCHF groups (Figure 8). The *p*-values obtained from the two-sample *t*-test <0.05 indicate a significant difference in the liver and skeletal muscle glycogen concentrations between the SD group and the HCHF group, with the HCHF group having higher liver and skeletal muscle concentrations compared to the SD group (Table 14).

### 3.8. Homeostatic Model Assessment for Insulin Resistance (HOMA-IR)

The HOMA-IR index was calculated in the SD group and HCHF group. The HOMA-IR value of the SD group was within the insulin-sensitivity range (<1.0). By comparing the SD group, the HCHF group showed a significantly higher HOMA-IR value, which was above the early IR range (>1.9) but lower than the overt IR range (>2.9). The *p*-value obtained from the Wilcoxon rank sum test was <0.05, indicating significant differences between the SD and HCHF groups (Table 15).

### 3.9. Plasma Insulin

**Figure 9:**
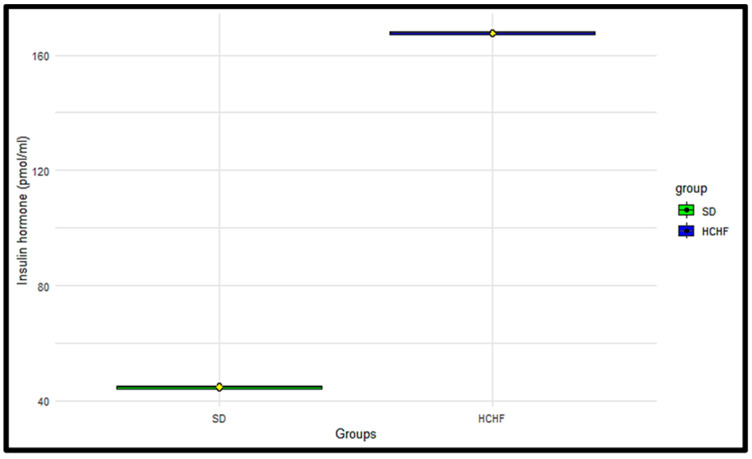
The terminal distributions of plasma insulin leptin concentrations in the SD and HCHF groups. The horizontal line within each box represents the median, while the box encompasses the interquartile range (IQR). The whiskers extend to the minimum and maximum values. The yellow diamond shape represents the mean. The black dots represent the individual measurements.

The plasma insulin levels were measured terminally in the SD and HCHF groups (Figure 9). The *p*-values obtained from the two-sample *t*-test < 0.05 indicated a significant difference in the plasma insulin concentrations between the SD group and the HCHF group, with the HCHF group having higher plasma insulin concentrations compared to the SD group (Table 16).

### 3.10. Plasma Ghrelin

**Figure 10:**
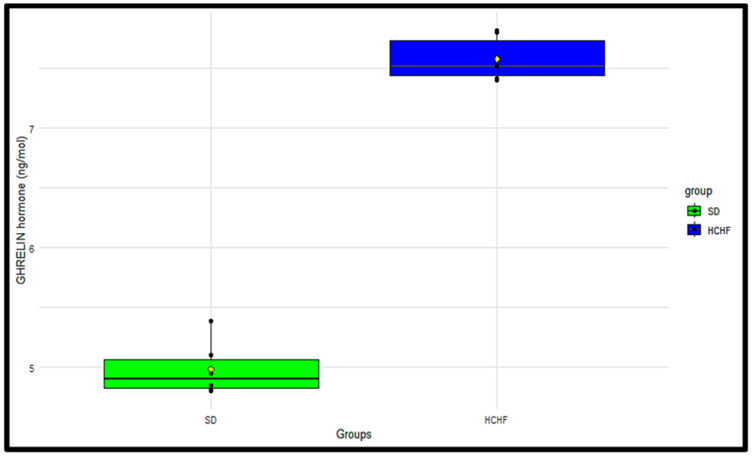
The terminal distributions of the plasma ghrelin concentrations in the SD and HCHF groups. The horizontal line within each box represents the median, while the box encompasses the interquartile range (IQR). The whiskers extend to the minimum and maximum values. The yellow diamond shape represents the mean. The black dots represent the individual measurements.

Plasma ghrelin concentrations were measured terminally in the SD group and the HCHF group (Figure 10). The *p*-value obtained from the two-sample *t*-test was <0.05, indicating a significant difference in the plasma ghrelin concentrations between the SD group and the HCHF group; the HCHF group had higher plasma ghrelin concentration compared to the SD group (Table 17).

### 3.11. Plasma Leptin

**Figure 11:**
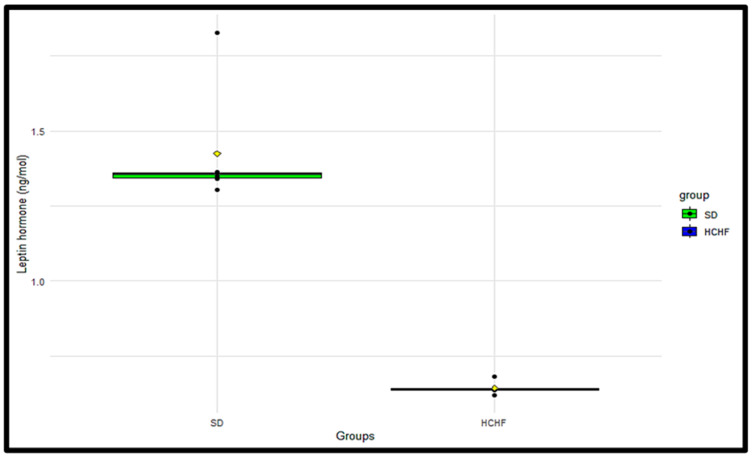
The terminal distributions of plasma leptin concentrations in the SD and HCHF groups. The horizontal line within each box represents the median, while the box encompasses the interquartile range (IQR). The whiskers extend to the minimum and maximum values. The yellow diamond shape represents the mean. The black dots represent the individual measurements.

The plasma leptin concentrations were measured terminally in the SD and HCHF groups (Figure 11). The *p*-value obtained from the Wilcoxon rank sum test was <0.05, indicating a significant difference in the plasma leptin concentrations between the SD group and the HCHF group, with the HCHF group having a lower plasma leptin concentration compared to the SD group (Table 18).

## 4. Discussion

Impaired glucose tolerance and impaired fasting glucose are two of prediabetes’ most noticeable characteristics [7]. These two conditions are often present prior to type 2 diabetes mellitus and are linked to moderate insulin resistance in the insulin-dependent tissues [7]. The development of insulin resistance can be attributed to obesity and the chronic consumption of high-calorie diets that contain trans fats, saturated fats, refined grains, and highly sweetened sugars [18,25,26]. A positive correlation between the development and progression of prediabetes in a male animal model and the consumption of a high-fat, high-carbohydrate diet has been established in our laboratory [18]. Most of the established prediabetic models involve male animals, raising concerns about the underrepresentation of females in clinical and pre-clinical research [26,27,28]. The National Institute of Health (NIH) has, however, encouraged the use of both sexes as study subjects and has taken into account sex as a biological factor, noting the possible harmful consequences of sex bias on human health [29]. Hence, our study sought to establish a diet-induced prediabetes female animal model. This study evaluated the effects of the chronic ingestion of a high-fat, high-carbohydrate diet supplemented with 15% fructose on parameters associated with prediabetes and the development of type 2 diabetes.

Glucose, a necessary fuel, must constantly be present in the blood in a sufficient quantity for every living organism [7]. A physiological range of 3.5–5.5 mmol/L should be maintained for blood glucose levels, including a post-prandial blood glucose level of <7.8 mmol/L and fasting blood glucose (FBG) below 5.6 mmol/L [30,31]. For those with normal glucose tolerance, in the post-prandial state, their blood glucose concentrations rise, and insulin is produced to promote glycogenesis and suppress glycogenolysis [32]. Subsequently, the normal physiological range for blood glucose levels is maintained, and the insulin levels return to normal. However, for those individuals with impaired fasting glucose and/or impaired glucose tolerance, the endogenous production of glucose is increased in the pre-prandial state and remains increased even after food consumption, which is a consequence of the impaired insulin-induced peripheral glucose uptake in insulin-sensitive tissues [32,33]. This explains the increased levels of insulin, post-prandial glucose concentration, fasting plasma glucose, and HOMA-IR in prediabetic individuals compared to normal glucose-tolerant individuals [34]. In the current study, we reported the fasting plasma glucose concentrations and the post-postprandial glucose concentrations after 2 h, as seen in Figure 2 and Figure 3, respectively, which were notably higher in the HCHF-fed group; however, it was only at week 36 that these values met the ADA criteria for the diagnoses of prediabetes. In this study, the model displayed indications of prediabetes, which, by the ADA criterion, are defined by the presence of impaired glucose tolerance (IGT) between 7.8 and 11.0 mmol/L and/or impaired fasting glucose (IFG) between 6.1 and 6.9 mmol/L [20]. Both of these are conditions of insulin resistance linked to the dysfunction of the pancreatic beta cells [7,35]. Our results corroborated with previous findings that also displayed significantly higher plasma glucose concentrations and 2 h post-prandial blood glucose levels in the prediabetic state compared to the non-prediabetic state [36].

Individuals with normal glucose tolerance are able to maintain homeostatic levels of plasma insulin [37]. However, in prediabetic conditions, there are higher insulin secretion, HbA1c, blood glucose concentration, and early insulin resistance levels than in normal glucose-tolerant conditions [7,38]. In the prediabetic condition, insulin fails to trigger a response in insulin-dependent tissues, such as the skeletal muscle; as a result, the accumulation of glucose in circulation promotes glycation with hemoglobin, which then causes the β-cells of the pancreas to react by producing more insulin in order to control the rise in blood glucose levels, leading to compensatory hyperinsulinemia [39,40]. This would eventually lead to the exhaustion of the pancreatic β cell and insulin insufficiency, which could manifest in the latter stages of type 2 diabetes mellitus [41]. Our findings showed that the HbA1c levels, as seen in Figure 7, HOMAR-IR index, as seen in Table 15 and plasma insulin concentrations, as seen in Table 16, were considerably higher in the HCHF-fed group compared to the SD-fed group. These results supported previous studies, which also displayed elevated HbA1c, HOMA-IR, and plasma insulin levels in prediabetic conditions compared to those in non-prediabetic conditions [42]. In the HCHF-fed group, the increased plasma insulin, impaired fasting glucose, and HOMA-IR values indicate insulin resistance from peripheral tissue against glucose absorption. Over the span of an erythrocyte’s 120-day life, a persistent rise in plasma glucose concentration, as in the case of insulin resistance, has been demonstrated to cause glucose-mediated non-enzymatic glycation of hemoglobin via the Amadori reaction, forming a permanently irreversible ketoamine linkage [43,44,45]. This supports the findings of the current study, which demonstrate that the ingestion of the HCHF diet leads to the elevated formation of glycated hemoglobin, as seen in Figure 7. This indicates that the extent and exposure of the erythrocytes to the circulating glucose levels may have resulted in the formation of HbA1c.

The hormone insulin has been demonstrated to modulate food intake, partly through ghrelin suppression, to regulate the levels of glucose entering the body [46]. The current study shows that ingesting the HCHF diet with fructose results in increased plasma ghrelin concentrations, as seen in Figure 10, and decreased plasma leptin, as seen in Figure 11; this is accompanied by consistently heightened food intake (Figure 4) regardless of the elevated plasma insulin concentration in these HCHF-fed animals. This indicates that the chronic consumption of this diet enhances appetite and causes an imbalance in the typical inverse insulin–ghrelin interaction [47]. Increased plasma ghrelin concentration and decreased plasma leptin concentrations in the presence of lowered insulin sensitivity eventually result in a consistent rise in plasma glucose concentration [46]; this supports our finding, as this occurrence is also seen in the HCHF-fed group. Earlier research has also demonstrated that high-fructose diets affect the control of appetite, and that intake of fructose causes reduced leptin and post-prandial suppression of ghrelin [48,49], thereby supporting the current study’s findings. Having a higher body mass index due to excess adiposity has been indicated to be the biggest risk factor for diabetes [50]. Research shows that long-term high-calorie food intake encourages excess adiposity, which raises waist circumference, body mass index, and cholesterol levels [27]. In the prediabetic stage, these have been identified as risk factors for the development of impaired glucose metabolism, insulin resistance, and cardiovascular illnesses [51,52]. The findings of the current study show that the HCHF-fed group had a significantly higher BMI than the SD-fed group, as seen in Figure 6, and this could be associated with the increased food intake levels observed in the HCHF diet-fed group.

There are moderate insulin resistance levels in prediabetes conditions. As a result, insulin continues to be effective because the pancreatic β-cells’ compensatory mechanism causes them to release more insulin into the blood; additionally, insulin is an anabolic hormone that encourages the body to store glucose as glycogen and fat [53,54]. In prediabetes, due to selective muscle insulin resistance and hyperinsulinemia, most of the consumed glucose is transferred to the liver, thus increasing hepatic glycogen synthesis [55]. The current study shows that the HCHF-fed group has significantly increased hepatic and skeletal muscle glycogen concentrations, as seen in Figure 8, compared to the SD-fed group. These results are consistent with the elevated insulin levels, as shown in Table 16, as insulin encourages the production of glycogen in these tissues. This observation is predicted in prediabetes conditions, given that insulin resistance is at moderate levels and pancreatic β-cells have not yet been exhausted, as is the case in overt type 2 diabetes mellitus.

In our previously established male model of prediabetes, the diagnostic parameters of prediabetes, as suggested by the ADA, were displayed within 20 weeks of ingesting the HCHF diet. This model closely resembles the condition of humans as it progresses. However, in the current study, the female rats only fully satisfied the three diagnostic parameters for prediabetes at 36 weeks of ingestion of the same HCHF diet. Research has demonstrated that men are diagnosed with type 2 diabetes at a younger age compared to women [4]. These findings relate to the results of our current study, which shows that the induction period takes much longer in female rats than in male rats, indicating that disease susceptibility differs in males and females, and this also shows that various aspects of energy balance and glucose metabolism differ between men and women, thus influencing their predisposition to type 2 diabetes mellitus [56]. We can also speculate that female sex hormones may play a role in the delayed onset of prediabetes. In this perspective, further studies need to be conducted on this female prediabetes model to elucidate the mechanisms specific to females that lead to the prolonged development of prediabetes in female rats, including conducting measurements on the female sex hormones during induction and termination. Measuring the expression levels of the estrogen receptor alpha can also provide a mechanism in which the estrogen signaling pathway is dysregulated or impaired, which will result in the estrogen hormone being unable to exert its protective effects, thus leading to metabolic dysfunction. The periodic measurement of the female sex hormones during the induction phase may demonstrate the negative effects of decreasing insulin sensitivity on the various pathways that rely on the supply of glucose to function effectively. Furthermore, the measurement of hormones and cytokines involved in glucose and lipid metabolism is required during the induction phase to accurately track and compare the pathophysiology changes that occur during induction and termination. This will provide a more in-depth understanding of the biochemical and homeostatic dysregulation that takes place in female models of prediabetes.

## 5. Conclusions

The majority of animal models of diabetes tend to skip the prediabetic stage, which is a massive setback in the understanding of its pathophysiology and the effective management of this condition. Developing a prediabetic animal model that closely represents the progression of diabetes in humans will help in tracking minute changes that occur at the initial stages of the condition, which eventually lead to major complications of diabetes. This study has successfully developed a female prediabetic rat model using a high-carbohydrate, high-fat diet. This model effectively replicates several important aspects of prediabetes observed in humans, such as impaired glucose tolerance and insulin resistance. Establishing this female animal model is an important contribution to research in prediabetes, as it can provide a reliable and reproducible system for studying the early stages of diabetes in females and can also be used to test potential therapeutic interventions. Additionally, it addresses the bias and underrepresentation of females in biomedical science research. The findings of this study demonstrate that a diet high in carbohydrates and fats can induce prediabetic conditions in female rats similar to humans, highlighting the critical role of dietary factors in the development of metabolic disorders in females, which is of great value in providing key advancements for deriving personalized treatment strategies for women and in understanding the influence of sex on health outcomes. We, therefore, suggest that this model can be used to further understand changes that occur during the progression of prediabetes to type 2 diabetes and can open new avenues for developing preventative methods for avoiding overt diabetes.

## Figures and Tables

**Table 1:** The composition of the high carbohydrate, high-fat diet (HCHF).

Ingredient	Incl. (%)	Mix
Maize	38.98	390.000
Palm Oil	20.99	210.000
Soya Full Fat	14.99	150.000
Wheat Gluten	6.50	65.000
Flour	6.00	60.000
Monodex	5.00	50.000
Sugar: White	5.00	50.000
Limestone	1.00	10.000
Dicalcium Phosphate	0.50	5.000
Vitamin Premix	0.35	3.500
Salt: Fine	0.30	3.000
Amino Acid–DL–Methionine	0.30	3.000
Mineral Premix	0.10	1.000
	**100.01**	**1000.50**

**Table 2:** The composition of the fat, protein, and carbohydrate contents of the standard diet (SD) and high-carbohydrate, high-fat diet (HFHC).

	SD (%Kcal/g)	HCHF (%Kcal/g)
Fats	15	30
Proteins	25	15
Carbohydrates	65	55

**Table 3:** The summary statistics of the fasting plasma glucose levels of the SD and HCHF groups.

Weeks	SD: Median (IQR) (mmol/L)	HCHF: Median (IQR) (mmol/L)	Wilcoxon Rank Sum Test *p*-Value
12	4.5 (4.4, 4.5)	4.5 (4.5, 4.5)	0.114
24	4.8 (4.8, 5.0)	5.4 (5.2, 5.5)	0.023
36	5.3 (5.0, 5.4)	5.8 (5.6, 6.0)	0.008

**Table 4:** The within-group comparisons of the fasting plasma glucose levels of the HCHF and SD groups.

Group	Friedman Test *p*-Value
SD	0.009
HCHF	0.002

**Table 5:** The summary statistics of the post-prandial glucose concentrations after 2 h in the SD and HCHF groups.

Weeks	SD: Median (IQR) (mmol/L)	HCHF: Median (IQR) (mmol/L)	Wilcoxon Rank Sum Test *p*-Value
12	4.6 (4.5, 4.6)	4.6 (4.5, 4.8)	0.541
24	5.3 (5.2, 5.5)	6.9 (6.4, 7.2)	0.005
36	5.4 (5.2, 5.4)	7.8 (7.8, 7.8)	0.004

**Table 6:** The within-group comparisons of the post-prandial glucose levels of the HCHF and SD groups.

Group	Friedman Test *p*-Value
SD	0.015
HCHF	0.002

**Table 7:** The summary statistics of the food intake levels of the SD and HCHF groups.

Weeks	SD: Median (IQR) (g)	HCHF: Median (IQR) (g)	Wilcoxon Rank Sum Test *p*-Value
12	22.0 (20.0, 23.0)	29.5 (29.0, 30.0)	0.004
24	23.0 (22.0, 24.0)	32.0 (30.0, 33.0)	0.005
36	36.0 (25.0, 27.0)	34.0 (32.0, 35.0)	0.005

**Table 8:** The within-group comparisons of the food intake levels of the HCHF and SD groups.

Group	Friedman Test *p*-Value
SD	0.003
HCHF	0.013

**Table 9:** The summary statistics of the SD and HCHF groups’ body weights.

Weeks	SD: Median (IQR) (g)	HCHF: Median (IQR) (g)	Wilcoxon Rank Sum Test *p*-Value
12	325.0 (324.0, 326.0)	346.5 (344.0, 347.0)	0.004
24	325.0 (324.0, 326.0)	353.0 (352.0, 354.0)	0.005
36	324.5 (324.0, 328.0)	359.0 (358.0, 361.0)	0.005

**Table 10:** The within-group comparisons of the body weights of the HCHF and SD groups.

Group	Friedman Test *p*-Value
SD	0.956
HCHF	0.002

**Table 11:** The summary statistics of the BMIs of the SD group and the HCHF group.

Weeks	SD: Median (IQR) (g/cm^2^)	HCHF: Median (IQR) (g/cm^2^)	Wilcoxon Rank Sum Test *p*-Value
12	0.932 (0.929, 0.934)	0.993 (0.986, 0.994)	0.004
24	0.931 (0.929, 0.934)	1.012 (1.009, 1.014)	0.005
36	0.930 (0.929, 0.940)	1.029 (1.026, 1.035)	0.005

**Table 12:** The within-group comparisons of the BMI of the HCHF and SD groups.

Group	Friedman Test *p*-Value
SD	0.717
HCHF	0.002

**Table 13:** The summary statistics of the glycated hemoglobin concentrations of the SD group and the HCHF group.

Variable	Group: Mean (Standard Deviation) [95% CI]		Independent Sample *t*-Test *p*-Value
	SD	HCHF	
Hb1Ac hormone	104.204 (1.551) [102.576, 105.832]	300.819 (3.654) [296.984, 304.654]	<0.001

CI: Confidence interval.

**Table 14:** The summary statistics of the liver and skeletal muscle glycogen concentrations of the SD group and the HCHF group.

Variable	Group: Mean (Standard Deviation) [95% CI]		Independent Sample *t*-Test *p*-Value
	SD	HCHF	
Liver glycogen	0.085 (0.004) [0.081, 0.088]	0.192 (0.013) [0.178, 0.206]	<0.001
Muscle glycogen	0.069 (0.002) [0.067, 0.072]	0.112 (0.011) [0.100, 0.124]	<0.001

CI: Confidence interval.

**Table 15:** The summary statistics of the HOMA-IR indices of the SD group and the HCHF group.

Variable	Group: Median (IQR)		Wilcoxon Rank Sum Test *p*-Value
	SD	HCHF	
HOMA-IR index	0.845 (0.840, 0.960)	3.150 (3.150, 3.150)	0.004

**Table 16:** The summary statistics of plasma insulin levels in the SD group and the HCHF group.

Variable	Group: Mean (Standard Deviation) [95% CI]		Independent Sample *t*-Test *p*-Value
	SD	HCHF	
Insulin hormone	44.700 (0.632) [44.036, 45.364]	167.617 (0.531) [(167.060, 168.174]	<0.001

CI: Confidence interval.

**Table 17:** The summary statistics of the plasma ghrelin of the SD group and the HCHF group.

Variable	Group: Mean (Standard Deviation) [95% CI]		Independent Sample *t*-Test *p*-Value
	SD	HCHF	
Plasma ghrelin	4.985 (0.226) [4.747, 5.222]	7.580 (0.186) [7.385, 7.775]	<0.001

CI: Confidence interval.

**Table 18:** The summary statistics of the plasma leptin of the SD group and the HCHF group.

Variable	Group: Median (IQR)		Wilcoxon Rank Sum Test *p*-Value
	SD	HCHF	
Plasma leptin	1.355 (1.340, 1.362)	0.640 (0.635, 0.642)	0.005

## Data Availability

The original contributions presented in the study are included in the article; further inquiries can be directed to the corresponding author/authors.
